# Fibrin Hydrogel Based Bone Substitute Tethered with BMP-2 and BMP-2/7 Heterodimers

**DOI:** 10.3390/ma8030977

**Published:** 2015-03-06

**Authors:** Lindsay S. Karfeld-Sulzer, Barbara Siegenthaler, Chafik Ghayor, Franz E. Weber

**Affiliations:** 1 Oral Biotechnology & Bioengineering, University Hospital, Division of Cranio-Maxillofacial and Oral Surgery and Center for Dental Medicine, University of Zurich, Plattenstrasse 11, Zurich CH-8032, Switzerland; E-Mails: lindsayksulzer@gmail.com (L.S.K.-S.); barabara.siegenthaler@usz.ch (B.S.); chafik.ghayor@usz.ch (C.G.); 2 Zurich Center for Integrative Human Physiology (ZIHP), University of Zurich, Zurich CH-8006, Switzerland; 3 CABMM, Center for Applied Biotechnology and Molecular Medicine, University of Zurich, Zurich CH-8006, Switzerland

**Keywords:** fibrin hydrogel, bone morphogenetic proteins, bone regeneration, bone substitute

## Abstract

Current clinically used delivery methods for bone morphogenetic proteins (BMPs) are collagen based and require large concentrations that can lead to dangerous side effects. Fibrin hydrogels can serve as osteoinductive bone substitute materials in non-load bearing bone defects in combination with BMPs. Two strategies to even further optimize such a fibrin based system include employing more potent BMP heterodimers and engineering growth factors that can be covalently tethered to and slowly released from a fibrin matrix. Here we present an engineered BMP-2/BMP-7 heterodimer where an N-terminal transglutaminase substrate domain in the BMP-2 portion provides covalent attachment to fibrin together with a central plasmin substrate domain, a cleavage site for local release of the attached BMP-2/BMP-7 heterodimer under the influence of cell-activated plasmin. *In vitro* and *in vivo* results revealed that the engineered BMP-2/BMP-7 heterodimer induces significantly more alkaline phosphatase activity in pluripotent cells and bone formation in a rat calvarial model than the engineered BMP-2 homodimer. Therefore, the engineered BMP-2/BMP-7 heterodimer could be used to reduce the amount of BMP needed for clinical effect.

## 1. Introduction

While bone autograft is the gold standard in healing critical-sized bone defects, there are significant drawbacks, including lack of supply and morbidity at the harvest site. Bone morphogenetic proteins (BMPs), a family of growth factors known to induce bone formation, have been supplied to defect sites in attempts to regenerate bone defects without an autograft. Since the discovery of BMPs in the 1960s [1], many studies with these growth factors have elucidated their critical role in bone induction and regeneration [2,3].

Although BMPs have demonstrated a high potential in healing bony defects, their delivery method is critical. Without a proper carrier, the half-life of BMPs is very short and retention at the desired site is difficult, preventing the sufficient concentration necessary to induce a cellular response [4,5]. Current BMP treatments require milligram doses, orders of magnitude higher than physiological levels [6]. Not only are these high concentrations expensive, but they can lead to undesired side effects [7]. A delivery system can localize the BMP and release it in a controlled fashion to limit ectopic bone formation in undesired locations [8,9]. Natural polymers, such as collagen, fibrin, and hyaluronic acid, ceramics, such as calcium phosphates and calcium cements, synthetic polymers, mostly polyesters, and composites of these materials are the main types of BMP delivery systems [4,5,8].

Many delivery systems incorporate the BMPs through non-covalent means, such as adsorption, ion complexation, or physical entrapment [10]. Alternatively, covalent mechanisms bind growth factors to the material in a more stable manner that can only be released through enzymatic or chemical cleavage or carrier degradation. Cells and environmental factors determine growth factor release, extending retention and limiting or eliminating the burst release found with many non-covalent systems that can lead to very high initial local concentrations. Since the growth factors are tethered to the material, immobilization further limits side effects due to diffusion to undesired areas. Moreover, this strategy enables spatial growth factor delivery and replicates physiological matrix interactions [11,12]. This ingrowth matrix concept is best realized by hydrogels and, in combination with growth factors, mimics the blood clot which forms upon injury [13,14]. One advantage of hydrogels is that covalently linked growth factors are presented to invading cells at the injured site. One major disadvantage of hydrogels is their low mechanical strength. Therefore, hydrogels alone can only be used for bone regeneration at non-load bearing sites like the cranium and need additional bone substitutes or fixation devices in load bearing sites like in the mandible or in long bones.

Fibrin is one of the extracellular matrix proteins that performs this critical function of regulating growth factor interplay with cells [15,16]. BMPs generally bind via heparin binding sites and have been shown to bind to fibrin (ogen) [17]. Fibrin is also a key component of wound healing, which is part of the process of bone regeneration [18]. Our lab has previously demonstrated that fibrin matrices with BMP-2 produced bone healing in various defect models, providing a replacement for autograft that simulates the natural fracture hematoma [14,19,20,21]. However, successful bone healing in these fibrin delivery systems with BMP-2 non-covalently incorporated, required high amounts of BMP-2 [22].

To address this issue with the delivery and retention of BMPs, we developed a covalent BMP binding strategy that utilizes an engineered BMP with an amino acid sequence that can be enzymatically attached to fibrin by using transglutaminase Factor XIII, an enzyme naturally found in blood clots [22]. Additionally, a plasmin degradation site enables cell-demanded growth factor cleavage. As also shown in other work, Factor XIII enzymatically crosslinks hydrogels and/or conjugates growth factors and bioactive peptides to fibrin or fibrin-like materials [23,24,25,26,27,28,29,30], by forming a bridge between the side chains of lysines and glutamines. By inclusion of a specific active glutamine sequence at the N-terminal of BMP-2, this growth factor was designed to attach to lysine residues present in fibrin. Denoted TG-BMP-2, this manipulated BMP attached to fibrin demonstrated superior results compared to native BMP-2 in small animal models [22].

In addition to tethering BMP to decrease the supplied effective amount of BMP, a second route we explored is increasing the potency of the delivered growth factor. Although there are 15 BMP variants, BMP-2 and BMP-7 have been shown to be particularly efficacious and have been commercially developed [3,31,32]. All BMPs consist of two identical polypeptide chains that are connected through disulfide bonds and thus referred to as homodimers. However, convincing evidence indicates that these factors function in sync to induce bone growth [33]. Moreover, multiple studies have demonstrated that the heterodimers, particularly BMP-2/7, have a greater activity, thus requiring a lower concentration for bone regeneration or improving bone growth outcome measures [6,34,35,36]. In the present study, we report on a recombinantly created heterodimer with one strand of TG-BMP-2 and the other of BMP-7, denoted as TG-BMP-2/BMP-7.

Here we characterize these engineered TG-BMP-2 and TG-BMP-2/BMP-7 growth factors *in vitro* and study their *in vivo* efficacy with a rat calvarial defect model in conjunction with a fibrin matrix.

## 2. Results

### 2.1. Heterodimer

**Figure 1:**
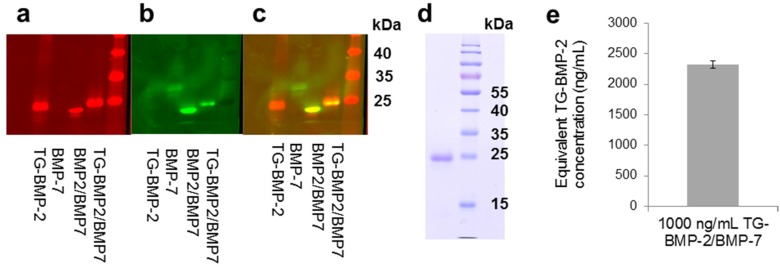
TG-BMP-2/BMP-7 heterodimer characterization. Western blot of the TG-BMP-2/BMP-7 heterodimer compared to TG-BMP-2, R&D Systems BMP-7 and R&D Systems BMP-2/BMP-7. (**a**) BMP-2 detection (**b**) BMP-7 detection and (**c**) overlay (**d**) PAGE of TG-BMP-2/BMP-7 shown next to molecular weight marker (**e**) The ALP activity of TG-BMP-2/BMP-7 is almost 2.5 times more active than TG-BMP-2.

The TG-BMP-2/BMP-7 heterodimer was successfully recombinantly produced through separately expressing and purifying the individual monomers and then refolding them together. Following refolding, the heterodimer was purified from all other components, including possible homodimers and misfolded proteins, through a sequence of steps: affinity, size exclusion, and reverse phase chromatography. Through the use of infrared secondary antibodies in a Western blot detecting BMP-2 and BMP-7, the heterodimer was confirmed by the overlap of the BMP-2 and BMP-7 signal (Figure 1a–c). Both polypeptide chains of the heterodimer should have the same migration in a non-reduced Western blot.

An SDS-PAGE verified that the TG-BMP-2/BMP-7 heterodimer is pure, showing only a single band (Figure 1d). Analysis of the activity of the heterodimer supported other researchers’ findings that the heterodimer is more active than homodimers. Although a non-glycosylated BMP-7 homodimer was not available for comparison, the heterodimer demonstrated an elevated activity almost 2.5 times greater than the TG-BMP-2 homodimer in a standard alkaline phosphatase assay (Figure 1e).

### 2.2. Functionality of Engineered Growth Factors

**Figure 2:**
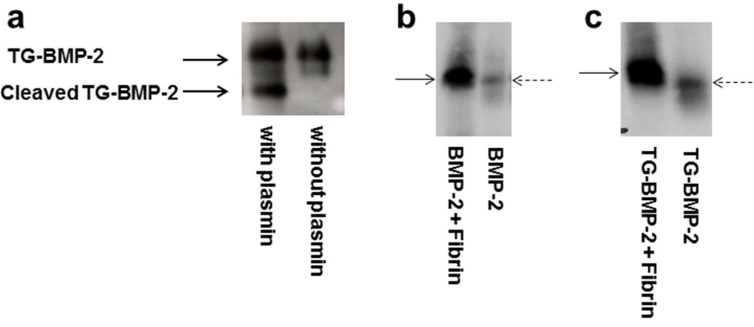
Functionality of plasmin and transglutaminase site. (**a**) Plasmin degradation of TG-BMP-2. TG-BMP-2 was incubated with plasmin or only buffer. Plasmin cleaves the TG-BMP-2. (**b**) and (**c**) Enzymatic digestion of growth factor alone and growth factor incorporated into a fibrin gel. (**b**) BMP-2 in fibrin digested by trypsin (**c**) TG-BMP-2 in fibrin digested by trypsin. Dashed arrow is the growth factor alone and solid arrow marks the fibrin material with the growth factor. The bands from the TG-BMP-2 incorporated into the fibrin have lower mobility than TG-BMP-2 alone, indicating that the TG-BMP-2 is covalently incorporated. In contrast, the BMP-2 has the same mobility alone as is incorporated into a fibrin gel.

Although a previous study in our laboratory indicated that the engineered TG-BMP-2 with a fibrin matrix improved bone growth *in vivo* [22], the current work characterizes and explores these growth factors and materials in greater depth. As part of this analysis, the functionality of both the plasmin cleavage site and the enzymatic attachment site was evaluated. By incubating TG-BMP-2 with plasmin and assessing the sample on a Western blot, the plasmin site was deemed to be functional. As shown in Figure 2a, TG-BMP-2 digested with plasmin shows two bands on a Western blot probing BMP-2, while TG-BMP-2 without plasmin displays only the full length growth factor.

If the transglutaminase attachment site also functions as designed, the TG growth factors covalently, not physically, bind to fibrin-based materials. To evaluate this covalent attachment, a similar experiment was performed as previously done with an engineered beta nerve growth factor [25] and vascular endothelial growth factor [26]. The BMP growth factors alone or incorporated in a fibrin-based gel were digested with trypsin. Covalently attached engineered growth factors would retain some fibrin fragments upon digestion and thus have a larger molecular weight than the growth factor alone.

As shown in Figure 2b, when a regular BMP-2 without the TG site was incorporated into a fibrin gel and digested with trypsin, it migrated the same on a Western blot as BMP-2 alone, indicating no covalent incorporation, as expected. However, the same experiment with TG-BMP-2 showed that the TG-BMP-2 that had been in the fibrin gel has a lower mobility on the Western blot, as shown in Figure 2c, meaning that it had a higher molecular weight and was covalently attached. Thus, the TG site in TG-BMP-2 is functional.

**Figure 3:**
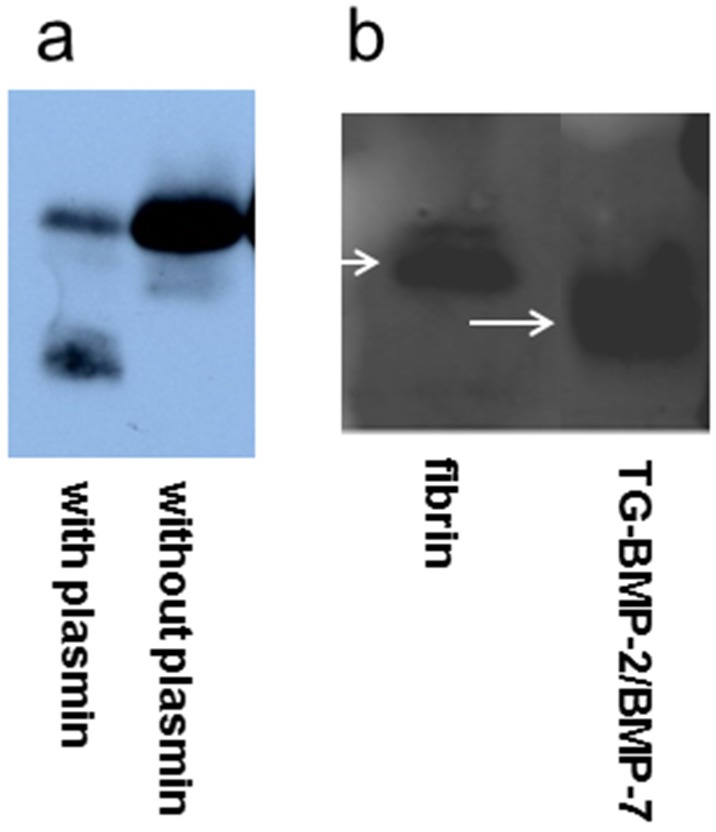
Functionality of TG-BMP-2/BMP-7 heterodimer indicated by probing BMP-2 (**a**) TG-BMP-2/BMP-7 was incubated with plasmin or only buffer. Plasmin cleaves the TG-BMP-2. (**b**) The heterodimer alone or contained in a fibrin gel was incubated with trypsin. Lower mobility of TG-BMP-2/BMP-7 in the fibrin gel indicates covalent incorporation.

The TG-BMP-2/BMP-7 heterodimer was also characterized to ensure that the cleavage and attachment sites are functional. Figure 3a shows that TG-BMP-2/BMP-7 digested by plasmin produced the cleaved protein. Incorporation of the heterodimer into fibrin gels followed by trypsin digestion showed a lower mobility of the attached growth factor and thus confirmed its functionality, as seen in Figure 3b.

**Figure 4:**
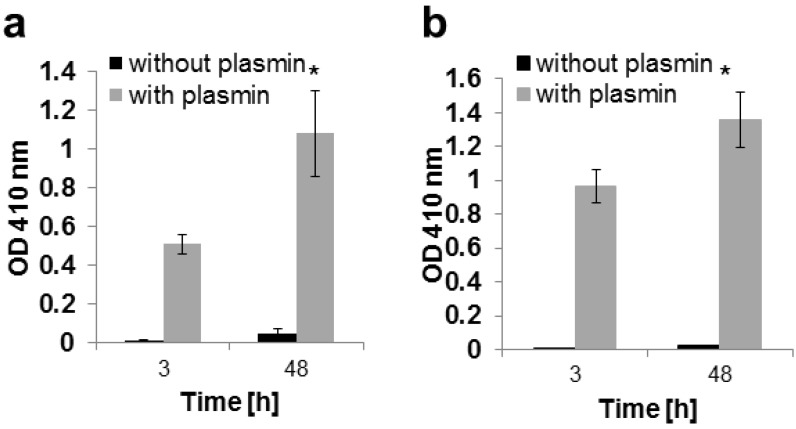
Growth factors are released and active after incorporation into fibrin gels and digested by plasmin as measured with an alkaline phosphatase assay and assessed at 410 nm. The activity for the plasmin-digested growth factor is significantly higher at 48 h than 3 h, * *p* < 0.05 (**a**) TG-BMP-2; (**b**) TG-BMP-2/BMP-7.

Another important aspect of these engineered growth factors is maintenance of activity after covalent attachment and release. As shown in Figure 4a,b, both TG-BMP-2 and TG-BMP-2/BMP-7 are active after incorporation into a fibrin gel and subsequently digested with plasmin. Without plasmin, no activity is detected since the growth factors are still connected to the gel and not in the assayed supernatant. Furthermore, there is a significantly higher activity for both engineered growth factors at 48 h compared to 3 h, indicating that the action of plasmin is releasing additional molecules over time.

### 2.3. In Vitro Release

**Figure 5:**
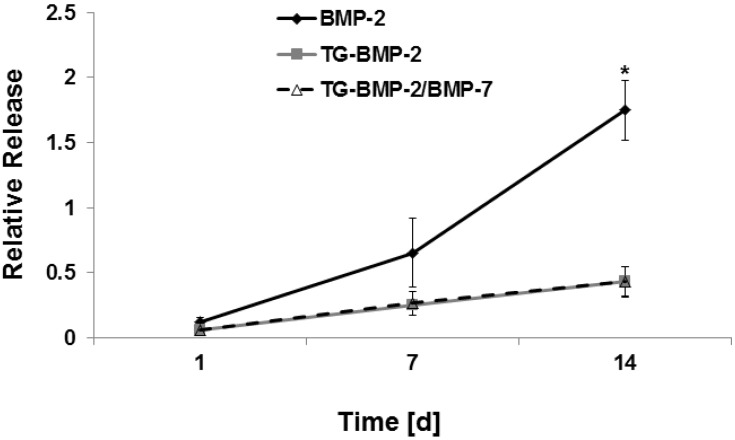
*In vitro* cumulative release from fibrin over days in DMEM normalized to the growth factor alone * *p* < 0.05.

*In vitro* release experiments supported the hypothesis that the tethered growth factors would have a longer retention time than the non-engineered BMP-2. The release of BMP-2, TG-BMP-2, and TG-BMP-2/BMP-7 incorporated into fibrin was assessed over the course of two weeks. Figure 5 illustrates that BMP-2 is released to a much greater extent by day 14 compared to TG-BMP-2 and TG-BMP-2/BMP-7. At day 14, the cumulative release of BMP-2 is significantly higher than the engineered growth factors by a factor of approximately 4.

### 2.4. In Vivo Testing

**Figure 6:**
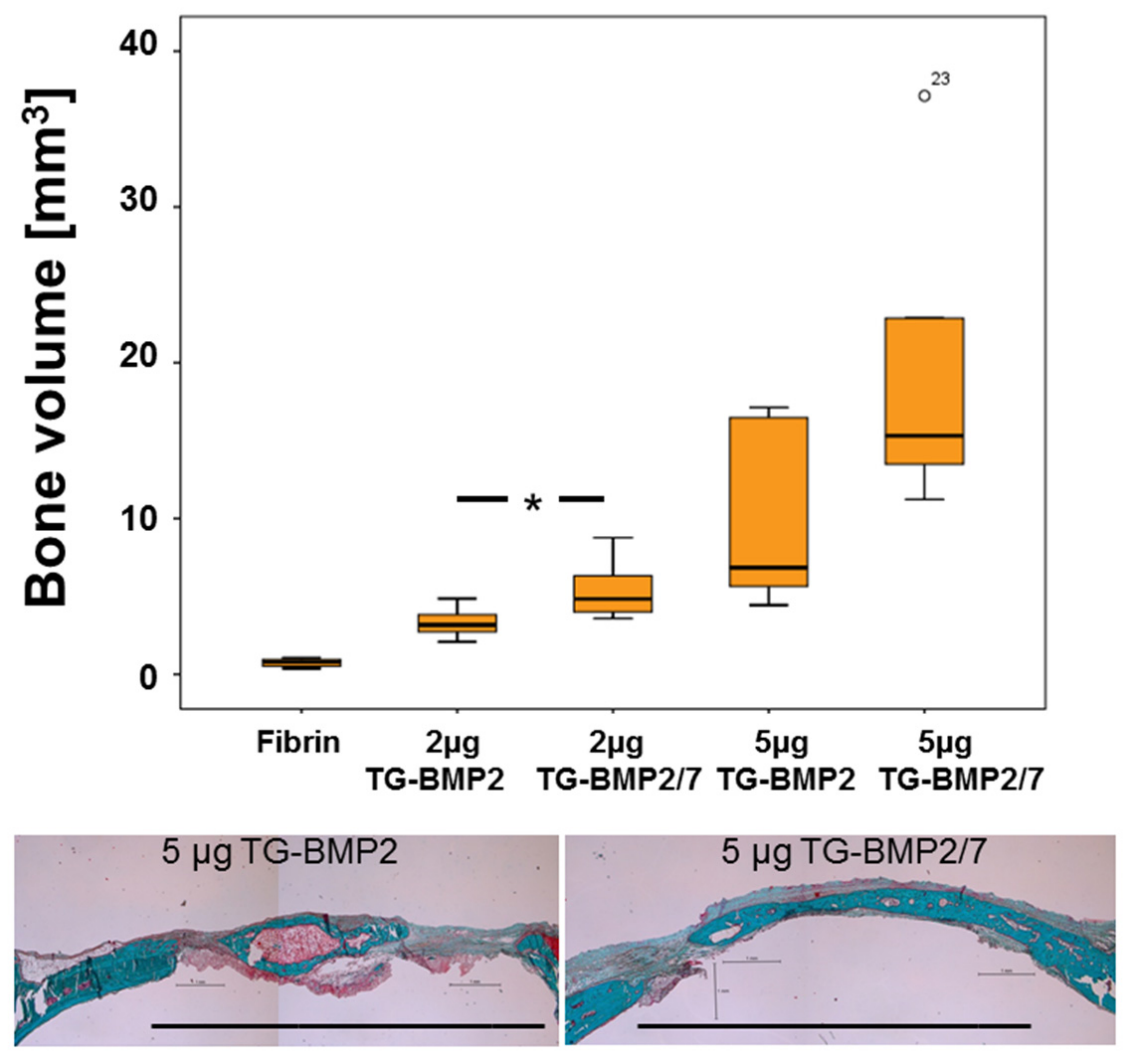
Healing of critical size calvarial defects in the rat. The ability of TG-BMP-2 and TG-BMP-2/BMP-7 in fibrin matrices to promote healing was examined in 8 mm defects in the rat, one per animal (n ≥ 5 for each group). The volume of the defect filled with calcified tissue after 35 days of healing with standard errors is shown along with representative Goldner trichrome stained histological sections of defects treated with 5 µg TG-BMP2 and TG-BMP-2/BMP-7 (lower panel). Original defect margins of 8 mm are indicated by the black bar. Bone is stained in green and osteoid in magenta. * indicates significance with a *p* < 0.05. ° indicates a data point outside of the boxplot.

*In vivo* efficacy of the homodimer TG-BMP-2 and the heterodimer TG-BMP-2/BMP-7 covalently attached to fibrin hydrogels was tested using 2 and 5 µg of the growth factors in a critical size calvarial bone defect model in rats. For both concentrations, the average formation of new bone in the defect margins was higher for TG-BMP-2/BMP-7 than for TG-BMP-2 at the evaluation point of 35 days. For the 2 µg growth factor concentration, TG-BMP-2/BMP-7 performed significantly better than TG-BMP-2, as shown in Figure 6. Bone quality of regenerated bone was assessed based on histologies (Figure 6 lower panel). The histologies revealed no adverse reaction on bone formation by either TG-BMP-2 or TG-BMP-2/BMP-7 although in the latter group, regenerated bone appeared to be more mature.

**Figure 7:**
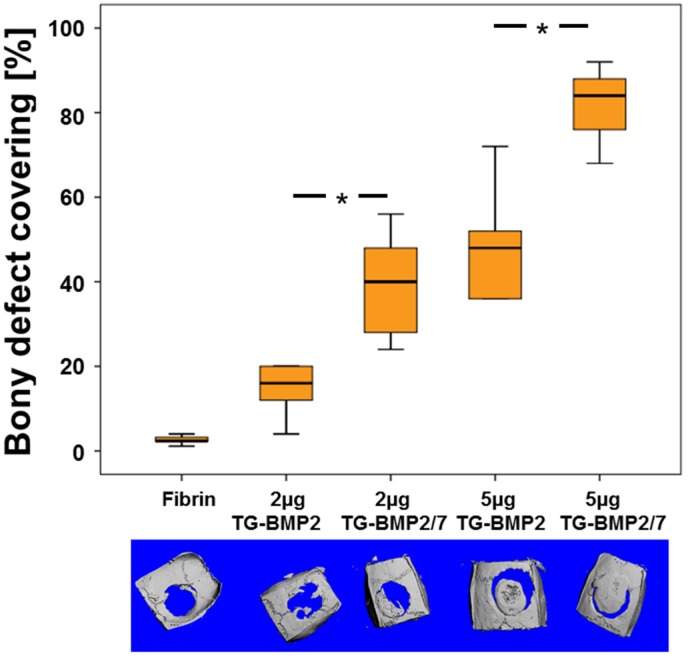
Bony covering of critical size calvarial defects in the rat. The ability of TG-BMP2 and TG-BMP-2/BMP-7 in fibrin matrices to promote healing was examined in 8 mm defects in the rat, one per animal (n ≥ 5 for each group). The area of the defect covered with calcified tissue after 35 days of healing with standard errors is shown along with µCT top views (lower panel). * indicates significance with a *p* < 0.05.

For both concentrations, the coverage of the defect with bone was significantly higher for TG-BMP-2/BMP-7 than for TG-BMP-2 at the evaluation point of 35 days (Figure 7). Therefore, the heterodimer TG-BMP-2/BMP-7 heals a critical size defect in the calvarial bone of rats more efficiently than the homodimer TG-BMP-2 at the same concentration.

## 3. Discussion

To improve the efficiency of BMP delivery for improving bone regeneration, a two-pronged approach was investigated. This strategy consisted of both increasing the BMP biological activity with heterodimers and sustaining the effect of the growth factors by tethering them to a fibrin delivery material.

Previous research demonstrated the elevated biological effect of BMP heterodimers over the respective homodimers [34,35,37,38], prompting the creation of an engineered TG-BMP-2/BMP-7 heterodimer. Other studies obtained BMP-2/BMP-7 growth factors via gene therapy [33], a fusion gene [6], mammalian cell culture [35], insect cell culture [39], and bacterial cell culture from a commercial source [36]. A patent on BMP heterodimers discusses various expression systems and processing methods [40]. The heterodimers used in the present work were created with *E. coli* culture to individually express and purify each monomer, followed by refolding the monomers and subsequently purifying the heterodimers. Although refolding must be performed in a separate step with bacterial cells, the low expense, high expression yields, and short culture times are distinct advantages for using this system. Purified TG-BMP-2 and BMP-7 monomers were combined in equimolar amounts for refolding using an already established protocol [41]. A series of centrifugation and concentration steps, followed by heparin affinity chromatography was used to separate the BMPs from all other constituents used in the refolding process. Size exclusion chromatography separated monomers from dimers and finally reverse phase chromatography discriminated between the correctly folded heterodimer and other dimers and higher order BMPs. This purification process yielded TG-BMP-2/BMP-7 that was pure and confirmed to be the heterodimer (Figure 1). Moreover, this molecule satisfied expectations with an activity approximately 2.5 times greater than the TG-BMP-2 homodimers. During this process formed TG-BMP-2 monomers have to be removed. This certainly lowers the yield of TG-BMP-2/BMP-7 production compared to TG-BMP-2 production and increases the cost of the heterodimeric compared to the homodimeric product.

As the other part of the overall approach, both the TG-BMP-2 homodimers and TG-BMP-2/BMP-7 heterodimers were thoroughly characterized to analyze their ability to be enzymatically attached and released from fibrin materials. These experiments (shown in Figure 2 and Figure 3) demonstrated that the designed features of these engineered growth factors were functional. Both the plasmin cleavage site and the enzymatic attachment site performed as expected. Moreover, these growth factors remained active after these processes (Figure 4). Although a prior study [22] had examined TG-BMP-2’s effect in multiple *in vivo* models, validation of the mechanism of attachment and release remained an important aspect to confirm.

The performance of these engineered growth factors was further verified through *in vitro* release studies. As shown in Figure 5, BMP-2 has significantly higher release than the TG growth factors by day 14. The increased BMP-2 release may only be detected after a number of days because the growth factors are physically trapped within the gel and may be released after some material degradation occurs. There is a much shallower release curve for the TG-BMP-2 and TG-BMP-2/BMP-7, but there is a small amount of release, which could stem from some of the growth factor molecules not being covalently attached due to limited accessible conjugation sites and enzyme efficiency. These unattached molecules are then released over a few days, similar to BMP-2. It is also interesting to note that the release curve of TG-BMP-2 is the same as that of TG-BMP-2/BMP-7, which concurs with the same attachment mechanism and similar size of these growth factors. In Figure 5, BMP-2 at day 14 has a relative release rate greater than one, even though one is the theoretical maximum release since this value corresponds to the activity of the maximum amount of growth factor in DMEM at 37 °C at the specified time point. However, these growth factors break down over time and lose activity at 37 °C [42] and it is possible that the BMPs within the gels are protected from degradation as compared to when alone in DMEM, thus raising the measured activity.

In a critical size defect model in rats, bony bridging of the defect was significantly higher at both concentrations and bone formation was significantly higher with 2 µg TG-BMP-2/BMP-7 than with the same amount of the homodimer TG-BMP-2. Since the release of both homo and heterodimers is almost identical, this improvement can be attributed to the increased activity of the heterodimer TG-BMP-2/BMP-7 compared to the homodimer of TG-BMP-2. This result validates the higher activity of the heterodimer *in vivo* and agrees with previous reports about unmodified constructs [6,34,35,36] where heterodimers of BMP-2/BMP-7 performed better than homodimers of BMP-2.

## 4. Experimental Section

### 4.1. BMP-2, TG-BMP-2, and TG-BMP-2/BMP-7 Production

BMP-2, TG-BMP-2, and TG-BMP-2/BMP-7 were recombinantly produced in our laboratory in E. coli in a process described previously [22,43]. The TG-BMP-2 homodimer and the TG-BMP-2/BMP-7 heterodimer included the tranglutaminase amino acid sequence along with a plasmin degradation site at the N-terminus. The extra amino acid sequence, M N Q E Q V S P L P V E L P L I K M K P H, is followed directly by the active BMP-2 sequence, amino acids 283–396 from the human sequence. The heterodimer contained one chain of TG-BMP-2 and another of BMP-7 with the amino acids 293–431 in the natural human sequence. The BMP-2 homodimer consists of active BMP-2 protein only. Briefly, the protein monomers were expressed individually in E. coli from a pET23a vector. After cells were expressed using IPTG induction, the cell membranes were ruptured using a French Press instrument. Inclusion bodies were dissolved in a Tris, EDTA, phenylmethylsulfonyl fluoiride (PMSF), and dithiothreitol (DTT) buffer and the resultant protein solution was acidified using glacial acetic acid and centrifuged. The supernatant was purified on a heparin binding HiTrap column (GE Healthcare, General Electric, Little Chalfont, UK) followed by gel filtration chromatography on a HiLoad 16/600 Superdex 200 column (GE Healthcare). Proteins were reduced using 0.1% DTT, dialyzed in 1% glacial acetic acid, lyophilized, and stored at −80 °C for later use. To refold the monomers into functional dimers, the proteins were incubated at 4 °C in a 50 mM Tris, pH 8.5, 1 M NaCl, 5 mM EDTA, 2% CHAPS (3((3-cholamidopropyl)-dimethylammonio)-propansulfonate), 2 mM reduced glutathione, and 1 mM oxidized glutathione buffer. For the heterodimers, TG-BMP-2 and BMP-7 monomers were combined in equimolar amounts. Following refolding, the homodimers were purified from the 10 mM HCl soluble portion on a HR 16/10 ProRPCTM reverse phase column (GE Healthcare) with a gradient of increasing acetonitrile with 0.1% trifluoroacetic acid (TFA), followed by isolation of the dimer with gel filtration chromatography on a HiLoad Superdex 75 column (GE Healthcare). The acidic soluble fraction of the TG-BMP-2/BMP-7 heterodimers was first purified on a heparin binding HiTrap column followed by the separation of the dimers from monomers using gel filtration chromatography on a HiLoad Superdex 75 column. Lastly, the heterodimers were purified from other dimers and high molecular weight aggregates using reverse phase chromatography on a RESOURCE RPC column (GE Healthcare). The protein solution was equilibrated in water with 1% TFA and elution occurred with an increasing gradient of acetonitrile with 1% TFA.

### 4.2. Plasmin Degradation

An amount of 10 µg TG-BMP-2 was diluted in 100 µL Tris buffered saline (TBS) and incubated overnight at 37 °C with 0.02 units plasmin (Sigma Aldrich) dissolved in 20 µL TBS. As a control, TG-BMP-2 was also incubated without plasmin.

### 4.3. BMP-2 Western Blot

Proteins were transferred to a PVDF membrane and blocked with 3% bovine serum albumin (BSA). An anti-BMP-2/4 primary antibody (R&D Systems) diluted 1:220 in 1% BSA was used to detect BMP-2 and TG-BMP-2. A polyclonal rabbit anti-goat IgG/HRP antibody (Dako) was diluted 1:2000 in 1% BSA. Lumi-Light Western Blot ECL reagents (Roche) were used to detect the chemiluminescent signal.

### 4.4. TG-BMP-2/BMP-7 Western Blot

To assure the formation of a heterodimer, overlapping detection of TG-BMP-2 and BMP-7 was assessed using orthogonal infrared secondary antibodies and imaging on an Odyssey CLx Infrared Imaging System (LI-COR Biosciences, Bad Homburg, Germany). After proteins were transferred to a PVDF membrane and blocked with 3% BSA, the first antibodies were incubated together overnight at 4 °C. TG-BMP-2 was detected with a goat anti-human BMP-2/4 primary antibody (R&D Systems) diluted 1:220 in 1% BSA and BMP-7 was identified with a mouse anti-human BMP-7 primary antibody (R&D Systems) diluted 1:3000 in 1% BSA. Both secondary antibodies, IRDye 680 RD donkey anti-goat IgG (LI-COR) and IRDye 800 CW donkey anti-mouse IgG (LI-COR) were diluted 1:15000 in PBS and incubated with the membrane for 1 h at room temperature. The BMP-2 and BMP-7 bands were detected at 700 nm and 800 nm wavelengths, respectively, on the Odyssey instrument. Overlapping bands indicated the heterodimer.

### 4.5. Fibrin Gel Formation

Fibrin gels were formed with 8 mg/mL fibrinogen (Enzyme Research Laboratories), 2 U/mL thrombin (Sigma Aldrich), 1 U/mL preactivated Factor XIII, and 2.5 mM Ca^2+^ in TBS, pH 7.4. Factor XIII (Fibrogrammin P, CSL Behring) was activated by incubating it for 30 min at 37 °C with 10% (v/v) thrombin (0.02 U/µL) in the presence of 25 mM CaCl_2_. Growth factors were included at the desired concentrations. Gels were allowed to fully form at 37 °C for at least 30 min.

### 4.6. Trypsin Degradation

To determine covalent attachment of growth factors, fibrin gels were incubated with TrypLE (Invitrogen), a recombinant trypsin-like enzyme.

### 4.7. Cell Culture

C2C12 cells (ATCC) were cultured with DMEM (Invitrogen) supplemented with 10% fetal bovine serum (Gibco), 1% 200 mM L-glutamine (Invitrogen) and 1% penicillin-streptomycin (10,000 units/mL pen, 10,000 ug/mL strep, Invitrogen). Cells were maintained at 37 °C in a humidified chamber with 5% CO_2_.

### 4.8. Alkaline Phosphatase Assay

Active BMP-2 and TG-BMP-2 were measured by incubating samples with C2C12 cells, a mouse myoblastic cell line, and measuring alkaline phosphatase (ALP) in cell lysates. After 5–7 days, media was removed; cells were washed in PBS, and incubated at room temperature for 30 min with 0.56 M 2-amino-2-methyl-1-propanol. Subsequently, cells were scraped from the plate and homogenized for 10 s. When cells were cultured in 96 well plates, 1% (v/v) Triton-X 100 was included along with the 0.56 M 2-amino-2-methyl-1-propanol and cells were not homogenized. The cell lysate supernatant was combined with an equal volume of 0.56 M 2-amino-2-methyl-1-propanol with 4 mM MgCl2 and 20 mM disodium p-nitrophenyl phosphate. Samples were heated at 37 °C for 10 min, NaOH was added to quench the reaction, and the optical density was read at 410 nm to measure alkaline phosphatase activity.

### 4.9. In vivo Rat Calvaria Defect

All animal procedures were approved by the Animal Ethics Committee of the local authorities (Kanton Zürich, 107/2012). Healthy female Sprague-Dawley (SD) rats were obtained from Charles River Laboratories. An 8 mm circular full thickness defect was created in the center of the calvaria using an electric burr. The bone was carefully removed, ensuring the dura were not disturbed. A quantity of 200 µL gels with a diameter of 8 mm with or without 2 µg or 5 µg TG-BMP-2 or TG-BMP-2/BMP-7 were formed in Teflon molds prepared the same day as the surgery and implanted into the defect. The wound was closed with surgical staples. Animals were sacrificed after 5 weeks and the calvaria were removed for analysis.

### 4.10. Microcomputed Tomography (µCT)

The calvaria, either submerged in 70% ethanol or embedded in methyl methacrylate (MMA), were imaged on a µCT 40 instrument (Scanco Medical AG, Brütisellen, Switzerland). The scanner was operated using a 70 kV energy setting, 145 µA intensity, a 300 ms integration time, medium resolution, two data averages, and an isotropic resolution of 30 µm. A 3-D representation was created to view the whole calvaria and identify the location of the defect. The new bone within the defect region was identified by contouring. Bone volume was calculated within this contoured area. A projection of the 3-D representation was used to determine the coverage of the defect with bone. Percentage of defect bridging was calculated within this area.

### 4.11. Histology

Samples were processed by a sequential water substitution by ethanol, infiltration by xylene followed by methyl-methacrylate. Polymerization was performed at 37 °C. Sections were prepared (6.5 μm) from the middle of the defects and stained with Goldner trichrome as reported earlier [44].

### 4.12. Statistics

Experiments were carried out independently at least three times. Release results are expressed as the mean ± SD and were compared by Student’s t-test. Histologies were evaluated by a Kruskal-Wallis Test followed by Mann-Whitney test for pairwise comparison. Results were considered significantly different for *p* < 0.05.

## 5. Conclusions

An engineered heterodimer of TG-BMP-2 and BMP-7 was produced and exhibited higher activity *in vivo* and *in vitro* than the TG-BMP-2 homodimer, but a similar slow release profile from fibrin hydrogels. With the application of a slow release TG-BMP-2/BMP-7 construct, the overall amount of BMP needed in the clinic could be decreased to provide a safer and more effective therapy.
